# Do Adolescents Use Substances to Relieve Uncomfortable Sensations? A Preliminary Examination of Negative Reinforcement among Adolescent Cannabis and Alcohol Users

**DOI:** 10.3390/brainsci10040214

**Published:** 2020-04-05

**Authors:** April C. May, Joanna Jacobus, Jennifer L. Stewart, Alan N. Simmons, Martin P. Paulus, Susan F. Tapert

**Affiliations:** 1 San Diego State University/University of California, San Diego Joint Doctoral Program in Clinical Psychology, San Diego, CA 92120, USA; jjacobus@health.ucsd.edu (J.J.); stapert@health.ucsd.edu (S.F.T.); 2 Department of Psychiatry, University of California, San Diego, San Diego, CA 92093, USA; ansimmons@health.ucsd.edu; 3 Laureate Institute for Brain Research, Tulsa, OK 74136, USA; JStewart@laureateinstitute.org (J.L.S.); mpaulus@laureateinstitute.org (M.P.P.); 4 Department of Community Medicine, University of Tulsa, Tulsa, OK 74104, USA

**Keywords:** cannabis, alcohol, adolescents, fMRI, interoception, negative reinforcement, cue reactivity

## Abstract

Alcohol and cannabis use are highly prevalent among adolescents and associated with negative consequences. Understanding motivations behind substance use in youth is important for informing prevention and intervention efforts. The present study aims to examine negative reinforcement principles of substance use among adolescent cannabis and alcohol users by pairing a cue reactivity paradigm with an aversive interoceptive stimulus. Adolescents (ages 15–17), classified as controls (CTL; *n* = 18), cannabis and/or alcohol experimenters (CAN+ALC-EXP; *n* = 16), or individuals meeting clinical criteria for cannabis and/or alcohol use disorder (CAN+ALC-SUD; *n* = 13) underwent functional magnetic resonance imaging during which they experienced an aversive interoceptive probe delivered via breathing load while simultaneously performing a cue reactivity paradigm. Participants also provided self-report ratings of how their substance use is positively or negatively reinforced. While experiencing the breathing load, CAN+ALC-SUD exhibited greater (*p* < 0.05) deactivation in the right amygdala, the left inferior frontal gyrus, and the left parahippocampal gyrus than CAN+ALC-EXP and CTL, who did not differ. Across all substance users, activation during the breathing load within the left parahippocampal gyrus negatively correlated with cannabis and alcohol lifetime use episodes and the left inferior frontal gyrus activity negatively correlated with lifetime alcohol use episodes. CAN+ALC-SUD reported experiencing more positive and negative reinforcement of using their substance of choice than CAN+ALC-EXP; both user groups reported higher levels of positive than negative reinforcement. Adolescents with a cannabis/alcohol use disorder demonstrate an altered response to interoceptive perturbations. However, adolescent cannabis/alcohol use does not appear to be driven by negative reinforcement, as viewing substance images did not dampen this response. Based on self-report data, the experience of positive reinforcement may be stronger for adolescents. Future studies should examine whether positive reinforcement contributes to adolescent substance use.

## 1. Introduction

Increased risk-taking behavior is characteristic of adolescence, a critical time period marked by significant physical, cognitive, and behavioral development [1]. A common risky behavior initiated in adolescence is the use of illicit substances. Among 12th graders, approximately 44% report having used cannabis and approximately 59% report having used alcohol in their lifetime [2]. Adolescent substance use can also evolve into a substance use disorder (SUD). For example, in 2018, 2.1% of adolescents aged 12–17 met criteria for cannabis use disorder, while 1.6% met criteria for alcohol use disorder [3]. Substance use during adolescence also increases future risk of experiencing adverse consequences related to use; early adolescent cannabis use may contribute to low educational or occupational attainment, as well as increased use and development of a use disorder in adulthood [4]. Similarly, youth who initiate drinking before age 15 are at increased risk of developing an alcohol use disorder within their lifetime compared to youth who remain abstinent until age 21 [5,6]. Given the increased risks associated with adolescent substance use, it is important to improve our understanding of the motivations behind these behaviors in order to inform SUD prevention and intervention efforts.

Altered interoceptive-related neural processing has been implicated in SUD in combination with emotion dysregulation and decision-making deficits, resulting in suboptimal behavioral adjustments and the propensity to continue drug use despite negative consequences [7,8,9,10,11,12]. To date, examination of the brain mechanisms involved in interoception and negative reinforcement has focused on adult SUD and little research has examined these concepts among adolescent substance users [13,14,15].

Interoception is a biological and psychological process by which somatosensory information from inside and outside of the body is filtered and integrated within the brain to produce an overall representation of the bodily state [16]. Anterior cingulate cortex (ACC), thalamus, frontal regions, and insular cortex (IC) are components of brain circuitry essential for processing and integrating bodily afferents to generate an overall representation of the body [16,17,18]. Afferent signals pass through thalamocortical pathways to IC to be integrated with sensorimotor activity and emotional information delivered by ACC and frontal regions such as inferior frontal gyrus (IFG) [16]. This process results in complex interoceptive feeling states or emotional awareness [18] and may lead to a bodily prediction error if the experienced state differs from the expected state [19,20,21,22]. Body prediction errors motivate individuals to engage in goal-directed behavior (e.g., substance use) and either approach or avoid stimuli (e.g., substance-related stimuli) with the aim of reestablishing equilibrium [23].

Among non-substance-using individuals, frontocingulate regions, including IFG and ACC, are thought to act as a regulatory system of behavioral reactions in response to aversive stimuli [24,25]. However, among individuals with SUD this regulatory system appears altered. For example, IFG and ACC blood oxygen-level dependent (BOLD) signal reductions in response to negative interoceptive stimuli have been found to characterize young adults transitioning to stimulant use disorders [8,26] while adolescent substance users have also demonstrated an increased IFG BOLD signal in response to a negative interoceptive stimuli [27]. In general, differing patterns of ACC, IFG, and IC activation have distinguished substance users from healthy individuals [28]. These frontocingulate deficits may be linked to reduced motivation to engage in behavioral changes to reestablish equilibrium despite feeling or sensing consequences of aversive bodily stimulation [29]. In addition to interoceptive processing, poor emotion regulation, an inability to effectively reduce arousal and cope with negative emotions has been implicated in adolescent substance use and requires similar brain regions [30]. The IFG and amygdala comprise a brain circuit involved in determining the emotional significance of an external stimulus and signaling the physiological, behavioral, cognitive, and affective responses necessary to minimize the impact of unpleasant stimuli [25,30,31,32,33]. 

One conceptualization of SUD, based on negative reinforcement principles, posits that individuals use drugs in order to alleviate uncomfortable feelings in general (e.g., emotional dysregulation, uncomfortable interoceptive states) [34,35]. For example, dysfunctional interoceptive processing may result in substance users seeking out and consuming drugs in order to reduce uncomfortable interoceptive states. Neuroimaging research suggests that drug cues activate brain regions similar to those activated by aversive interoceptive stimuli; cannabis cues elicit activation in parahippocampal gyri and various frontal regions among non-treatment-seeking cannabis-using adolescents [36]. Adolescents who primarily use alcohol also demonstrate an exaggerated neural response within frontal regions including IFG, parahippocampus, amygdala, and posterior cingulate in response to cue images [37]. Accordingly, the present study pairs an aversive interoceptive stimulus with a cannabis and alcohol cue reactivity task during functional magnetic resonance imaging. This pairing is viewed as a proxy for negative reinforcement, allowing for the examination of whether the rewarding effects of substance images dampen the negative experience of the breathing load. Specifically, we posit that viewing rewarding drug-relevant cues will dampen the interoceptive BOLD response observed in adolescent substance users while experiencing an aversive interoceptive stimulus.

An inspiratory breathing load can be used as an aversive stimulus to induce a negative interoceptive state [38] and has previously been tested among young adult [8,39], adult [40], and adolescent substance users [27] as well as matched controls. While experiencing the breathing load, young adults with problem stimulant use show lower IFG, IC, and ACC activation compared to individuals who no longer use stimulants as well as non-using controls [8,39]. Similarly, adults with a significant history of methamphetamine use currently meeting criteria for a methamphetamine use disorder also show lower IC and ACC during the breathing load [40]. Despite these differences in brain activation, groups did not differ in their subjective ratings of the breathing load experience. Overall, the reduced activation seen in regions implicated in interoceptive processing is conceptualized as an overall diminished ability to regulate when one does not feel well, and that this inability contributes to continued substance use despite negative consequences. To date, only one study has utilized an inspiratory breathing load with adolescent substance users; these results revealed an overactivation in interoceptive regions. This inconsistent finding suggests that alterations in interoceptive processing may differ as a function of age, type of substance used, or amount of substance used. 

The current study is the first to pair an aversive interoceptive stimulus with a cue reactivity paradigm to examine the role of negative reinforcement in substance use. In addition, the sample of the present study includes adolescents (ages 15–17) who report cannabis and alcohol use with and without use disorders. This will allow for the examination of negative reinforcement and interoceptive-related neural responses within diagnostically subthreshold adolescent substance users to investigate whether altered processing is simply a consequence of use or unique to adolescents experiencing functional impairments related to use (i.e., adolescents with use disorder diagnoses).

Participants included adolescents meeting criteria for either cannabis and/or alcohol use disorder, adolescents who use cannabis and alcohol but do not meet diagnostic criteria (experimenters) and healthy comparison participants. On the basis of prior work, it was hypothesized that substance users meeting diagnostic criteria compared to controls would show: (1) increased neural activation in response to the breathing load across all conditions of the cue task in brain regions involved in interoceptive processing, such as IC, ACC, and IFG, as well as regions implicated in emotion regulation, including amygdala and parahippocampal gyrus [27]; (2) increased striatal response while viewing substance images across all breathing load conditions, reflecting heightened reward responsivity to substance cues [41,42]; and (3) a blunted interoceptive neural response to the breathing load when paired with substance images (cannabis and alcohol images) suggesting exposure to a conditioned drug stimulus may help modulate reactions to internal and aversive states similar to negative reinforcement principals of drug use behavior. Additionally, adolescent substance users who did not meet criteria for SUD, referred to as “experimenters”, were included to explore whether neural differences are more pronounced in adolescent substance users who endorse substance use-related functional impairment (i.e., adolescents meeting criteria for SUD) than those who do not. Therefore, it was hypothesized that experimenters would demonstrate a neural response more similar to controls than those meeting substance use disorder criteria, suggesting that impaired brain responses are a consequence of more severe use symptomatology.

## 2. Materials and Methods

### 2.1. Participants

Adolescent participants (*n* = 47, ages 15–17) were recruited through local high schools by flyers that advertised an adolescent neuroimaging research study consisting of a clinical interview and neuroimaging session. The University of California San Diego Human Research Protections Program approved the study protocol. Adolescent participants provided assent and informed consent was obtained from one parent or legal guardian prior to study enrollment. Participants were excluded if they endorsed any of the following: (1) lifetime Diagnostic and Statistical Manual (DSM-5) of Mental Disorders psychiatric disorder (other than substance use disorder, SUD); (2) current use of psychoactive medications; (3) history of major neurological or medical disorder; (4) head injuries or loss of consciousness > 5 min; (5) irremovable metal in body; (6) pregnancy; (7) non-correctable vision or hearing problems; (8) premature birth or prenatal alcohol/drug exposure; (9) left handedness; or (10) claustrophobia. Eligible participants received financial compensation for their participation. 

The final sample consisted of 18 controls with very minimal histories of substance use (CTL; cannabis/alcohol maximum lifetime use episodes of 3 each, nicotine maximum lifetime use episodes of 10; 13M, 5F), 16 cannabis and alcohol experimenters (CAN+ALC-EXP; 12M, 4F), and 13 who met criteria for cannabis and/or alcohol use disorder (CAN+ALC-SUD; 9M, 4F). SUD group classification required a report of cannabis or alcohol use within the past three months, current endorsement of 2 or more DSM-5 SUD criteria for either cannabis or alcohol, and fewer than 15 lifetime uses of other drugs except for nicotine (see Table 1 for diagnostic details). On average, CAN+ALC-SUD participants reported 467 lifetime cannabis uses and 131 lifetime alcohol uses. CAN+ALC-EXP group classification required a report of no substance use history other than alcohol, cannabis, or nicotine, and no current or lifetime endorsement of DSM-5 SUD criteria. CAN+ALC-EXP reported significantly less cannabis (*t*(12.48) = −5.31, *p* < 0.001) and alcohol use (*t*(12.28) = −3.12, *p* < 0.009) than SUD but significantly more use of these substances than CTL (cannabis: *t*(15) = −3.46, *p* = 0.003; alcohol: *t*(15.06) = −4.29, *p* = 0.001) (see Table 1).

### 2.2. Clinical Interview

The clinical interview consisted of the Semi-Structured Assessment for Drug Dependence and Alcoholism (SSADDA; [43]) to assess for the presence of SUD and the Customary Drinking and Drug Use Record (CDDR) [44] to capture quantity of lifetime substance use, age of first use, and last substance use. Participants provided demographic information and a battery of self-report measures to assess characteristics related to SUD including the UPPS Impulsive Behavior Scale [45], the Multi-Dimensional Assessment of Interoceptive Awareness (MAIA) [46], and the Michigan Nicotine Reinforcement Questionnaire (MNRQ) [47]. The MNRQ was modified to assess negative reinforcement principles related to users’ substance of choice rather than nicotine. Each participant was asked to indicate their drug of choice (cannabis, alcohol) and answer the MNRQ questions regarding their experiences with that drug rather than nicotine. The specific questions and scale were not altered.

### 2.3. Neuroimaging Procedures

Participants were asked to abstain from substance use for at least 72 h prior to their fMRI session as confirmed by combination of self-report, breathalyzer, and urine toxicology screens. A positive result for any substance other than cannabis excluded individuals from the study. Acute cannabis use is difficult to determine by examination of urinary metabolites and therefore use within the past 72 h is possible; however, all participants self-reported abstaining for the 72 h prior to the appointment and only 5 (4 CAN+ALC-SUD, 1 CAN+ALC-EXP) participants were positive for THC on the day of testing, which could reflect use from up to four weeks prior given the regularity of their use history.

The Cue Breathing fMRI paradigm paired a cue reactivity task with anticipation and experience of an unpleasant interoceptive stimulus, an inspiratory breathing load. Each participant received either a cannabis or alcohol version of the task, depending on their reported primary substance of choice. For the cue reactivity task, participants were presented with images of substances (cannabis or alcohol), comparison images consisting of closely matched objects resembling the substance images (e.g., dried leaves resembling cannabis, non-alcoholic beverages), or scrambled versions of the substance and comparison images where the object in the image was unidentifiable. CTL viewed the same version of the task (cannabis or alcohol) as an age-matched substance-using participant. While viewing each image, participants were asked to indicate whether they disliked, felt neutral, or liked the image. Participants provided ratings using the first three buttons of a four-button box and saw a red box appear on screen to confirm their selected answer.

Participants wore a nose clip and respired through a mouthpiece with a non-rebreathing valve (2600 series, Hans Rudolph). The breathing equipment was attached to the scanner head coil using Velcro straps to help hold the mouthpiece in position and eliminate the need for participants to contract their mouth muscles. The mouthpiece connected to a hose that allowed for an inspiratory resistance load of 40 cmH_2_O/L/s to be attached. This breathing load consisted of a Plexiglas tube with a sintered bronze disk inside that partially limited airflow thereby producing a resistance load. A breathing load of 40 cm H_2_O/L/s was selected based on previous research which has demonstrated that this load alters subjective symptoms without significantly affecting CO_2_ or O_2_ levels, and thereby does not impact the BOLD signal [48,49]. Prior to the scan, participants completed a training session during which they were introduced to the breathing equipment and practiced the task. Individuals experienced increasing levels of restriction up to the target load of 40 cm H_2_O/L/s. The breathing load was described as feeling like “you are breathing through a straw” and participants were instructed to continue to breathe normally while experiencing the restriction. While in the scanner, participants experienced the breathing load at various times throughout the task for approximately 40 s at a time. Each block of images began with one null trial that lasted for 6 s. During this time, participants saw either a yellow or grey fixation screen. Yellow indicated there was a 1 in 4 (25%) chance of experiencing the breathing load during the next block of images. Alternatively, a grey fixation screen indicated there would be no chance of experiencing the breathing restriction during the upcoming block of images. Each null trial was followed by 6 pictures of the same type (substance, neutral, or scrambled) presented one at a time for 4 s each.

**Figure 1:**
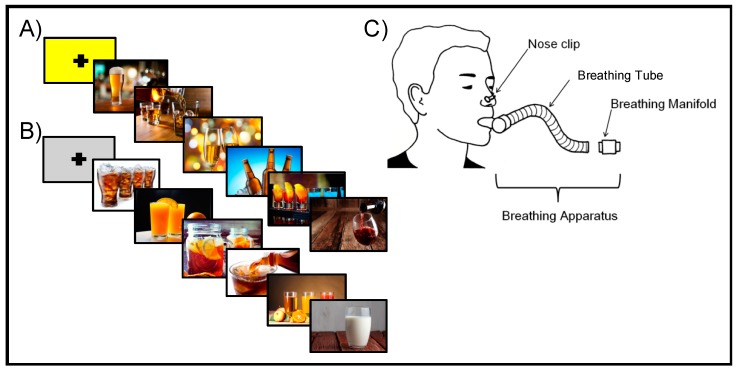
Depiction of the cue reactivity paradigm paired with interoceptive breathing load. (**A**) A yellow fixation screen is presented to the participant, indicating that there is a 1 in 4 chance they will experience the breathing load during the upcoming block of pictures. The fixation screen is immediately followed by 6 images—in this case, alcohol-related cue images. (**B**) A grey fixation screen is presented to the participant indicating that there is no chance they will experience the breathing load during the upcoming block of pictures. The fixation screen is immediately followed by 6 images—in this case, substance-matched comparison images. (**C**) Each participant wears the breathing apparatus while in the fMRI machine. They wear a nose clip to ensure they breathe through the tube only and a breathing manifold is attached at the end of the tube for periods of 40 s as indicated by the paired cue reactivity task.

There was a total of 9 task conditions: anticipation neutral images, anticipation substance images, anticipation scrambled images, breathing load neutral images, breathing load substance images, breathing load scrambled images, neutral images only, substance images only, and scrambled images only. Trials during which neutral or scrambled images were presented without the anticipation or experience of the breathing load were combined into a baseline condition. This resulted in 5 conditions of interest: (1) *baseline:* neutral and scrambled images with no anticipation or breathing restriction; (2) *anticipation neutral images:* blocks of neutral images preceded by a yellow fixation screen during which the participant did not actually experience the breathing load; (3) *anticipation substance images:* blocks of substance images preceded by a yellow fixation screen during which the participant did not actually experience the breathing load; (4) *breathing load neutral images:* blocks of neutral images preceded by a yellow fixation screen during which the participant did experience the breathing load; (5) *breathing load substance images:* blocks of substance images preceded by a yellow fixation screen during which the participant did experience the breathing load (see Figure 1).

Prior to the scan, participants underwent a training session to learn the task and become familiar with the breathing equipment. This ensured that participants would be able to complete the task within the scanner. Immediately after the scan, participants provided ratings of their in-scanner experience with the breathing load using a Visual Analog Scale (VAS). Participants rated the breathing load for pleasantness, unpleasantness, and intensity using a 10 cm scale ranging from ‘not at all’ to ‘extremely’. After the scan, participants used the same VAS to rate their in-scanner experience of the breathing load.

### 2.4. Neuroimaging Data Acquisition

The cue reactivity paradigm was presented during one fMRI scan sensitive to blood oxygenation level-dependent (BOLD) contrast using a Signa EXCITE (GE Healthcare, Chicago, IL, USA) 3.0 Tesla scanner (T2*-weighted echo planar imaging (EPI) scans, TR = 2000 ms, TE = 30 ms, FOV = 24 cm (squared), 64 × 64 × 40 matrix, forty 3.0 mm axial slices with an in-plane resolution of 3.75 × 3.75 × 3 mm, flip angle = 90 degrees, 420 whole-brain acquisitions). For anatomical reference, a high-resolution T1-weighted image (spoiled gradient recalled [SPGR], TR = 8 ms, TE = 3 ms, slices = 172, FOV = 25 cm approximately 1 mm^3^ voxels) was obtained.

### 2.5. Neuroimaging Data Analysis

#### 2.5.1. Individual-Level Processing

All neuroimaging data was processed using the Analysis of Functional NeuroImages (AFNI) software package [50]. Following data acquisition, GE slices were reconstructed into AFNI BRIK format. Baseline volume for 3D registration was constructed using the largest temporal region containing the fewest voxel-wise outliers. Data was aligned to the baseline image using all other time points in dx, dy, dz, and roll, pitch, and yaw directions. The functional EPI underwent automatic coregistration to the high-resolution anatomical image and each alignment was manually inspected for each dataset. New outliers were generated for the volume-registered dataset based on whether a given time point greatly exceeded the mean number of voxel outliers for the time series. Six motion regressors (dx, dy, dz and roll, pitch, and yaw), a baseline and linear drift regressor, and nine task-related regressors (trials for anticipation neutral images, anticipation substance images, anticipation scrambled images, breathing load neutral images, breathing load substance images, breathing load scrambled images, neutral images only, substance images only, and scrambled images only) were convolved with a modified hemodynamic response function. The baseline condition, during which there was no cue or experience of the breathing load, served as the baseline for this analysis. A Gaussian Spatial Filter (6 mm full width-half maximum) was used to spatially blur data to account for anatomical differences. Automated transformations were applied to anatomical images and EPIs were subsequently transformed into Montreal neurological institute (MNI) space. Percent signal change (PSC) was determined by dividing each regressor of interest (anticipation neutral images, anticipation substance images, breathing load neutral images, breathing load substance images) by the baseline regressor and multiplying by 100.

#### 2.5.2. Group-Level Analysis

A linear mixed-effects (LME) analysis (r-project.org) was performed to examine group differences in brain activation. Participants were treated as random effects, while group (CAN+ALC-SUD, CAN+ALC-EXP, CTL), interoceptive condition (no breathing load [anticipation], breathing load), and image type (neutral, substance) were treated as fixed effects. PSC from baseline (trials consisting of neutral and scrambled images and no chance or experience of the breathing load) was the dependent variable. The group main effect was examined to identify differences between CAN+ALC-SUD, CAN+ALC-EXP, and CTL across breathing load and cue image type conditions. The group by image type interaction was conducted to examine group differences while viewing substance images across all interoceptive conditions. The group by interoceptive condition interaction was examined to test hypotheses involving anticipation and receipt of the aversive interoceptive breathing load in CAN+ALC-SUD and CTL. The group by interoception by image type interaction was of interest because it allowed for examination of whether substance users show a blunted response to the aversive interoceptive stimuli when paired with the rewarding substance images. To guard against identifying false-positive areas of activation, a threshold adjustment method was applied using AFNI programs 3dFWHMx and 3dClustSim with the auto-correlation function (acf). The 3dClustSim identified a minimum cluster volume of 1280 µL (20 contiguous voxels) corresponding to a per-voxel *p*-value of 0.002 (bi-sided, NN = 3) to result in a voxel-wise probability of *p* < 0.05 (two-sided) corrected for multiple comparisons.

## 3. Results

### 3.1. Subject Characteristics

Groups did not differ in terms of demographics, including age (*F*(2, 44) = 1.27, *p* = 0.290), education (*F*(2,43) = 0.956, *p* = 0.392), racial (*χ*^2^(8) = 9.043, *p* = 0.339) and ethnic (*χ*^2^(2) = 0.10, *p* = 0.953) makeup, and gender distribution (*χ*^2^(2) = 0.37, *p* = 0.830); each group had more males than females. Moreover, there was no difference in subjective self-reported unpleasantness (*F*(2,44) = 0.432, *p* = 0.652) or intensity (*F*(2,44) = 2.68, *p* = 0.08) of the breathing load and the groups did not differ on self-reported interoceptive awareness and impulsivity. However, CAN+ALC-SUD compared to CAN+ALC-EXP reported higher levels of positive and negative reinforcement on the MNRQ. Additionally, both user groups reported higher levels of positive reinforcement than negative reinforcement on the MNRQ (see Table 2).

### 3.2. Neuroimaging Results

No clusters met the thresholding requirement of 20 voxels for the main effect of group, the group by image type interaction, or the three-way group by interoceptive condition by cue image type interaction.

#### 3.2.1. The Group by interoception interaction

Four brain regions survived thresholding: the right amygdala, the left IFG, the right posterior cingulate, and the left parahippocampal gyrus. (see Table 3). All interactions remained significant after controlling for lifetime nicotine use.

**Figure 2:**
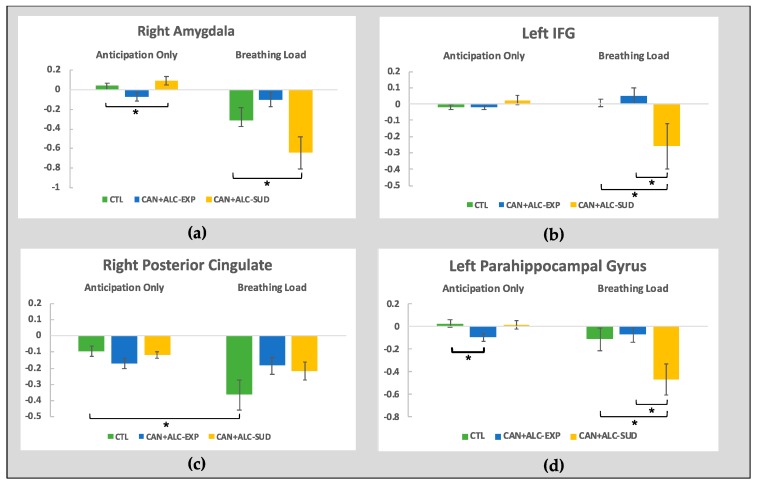
Neuroimaging results from the group by interoception condition interaction in (**a**) the right amygdala; (**b**) the left inferior frontal gyrus; (**c**) the right posterior cingulate; and (**d**) the left parahippocampal gyrus. * indicates significant differences.

The right amygdala. A significant interaction within the right amygdala (*F*(2,44) = 7.58, *p* = 0.001, partial *η*^2^ = 0.256) was examined. Here, groups significantly differed in activation for the anticipation only condition (*F*(2, 44) = 4.28, *p* = 0.02, partial *η*^2^ = 0.16) with CAN+ALC-SUD showing significantly greater activation than CAN+ALC-EXP (*M* = 0.16, *SE* = 0.06, *p* = 0.02). Groups also significantly differed during the breathing load condition (*F*(2, 44) = 4.59, *p* = 0.02, partial *η*^2^ = 0.17) with CAN+ALC-SUD showing lower activation than CAN+ALC-EXP (*M* = 0.54, *SE* = 0.18, *p* = 0.004). CTL did not significantly differ from either user group during either condition (see Figure 2).

The left inferior frontal gyrus. Within the left IFG (*F*(2,44) = 5.66, *p* = 0.006, partial *η*^2^ = 0.21), groups did not differ during anticipation (*p* = 0.28) but did during the breathing load (*F*(2,44) = 4.62, *p* = 0.015, partial *η*^2^ = 0.17). CAN+ALC-SUD exhibited lower activation than both CAN+ALC-EXP (*M* = 0.27, *SE* = 0.11, *p* = 0.049) and CTL (*M* = 0.27, *SE* = 0.11, *p* = 0.049), who did not differ from one another (see Figure 2).

The right posterior cingulate. An interaction within the right posterior cingulate (*F*(2,44) = 4.11, *p* = 0.02, partial *η*^2^ = 0.16) was driven by a significant effect of condition for CTL only (*F*(1,17) = 11.22, *p* = 0.004, partial *η*^2^ = 0.39) with greater deactivation while experiencing the breathing load; no simple main effect for group was seen in this region.

The left parahippocampal gyrus. Within the left parahippocampal gyrus (*F*(2,44) = 6.14, *p* = 0.004, partial *η*^2^ = 0.22), groups significantly differed during the anticipation condition (*F*(2,44) = 3.98, *p* = 0.02, partial *η*^2^ = 0.15) and during the breathing load trials (*F*(2,44) = 4.23, *p* = 0.02, partial *η*^2^ = 0.16). Specifically, during anticipation only trials, CTL exhibited significantly greater activation than CAN+ALC-EXP (*M* = 0.12, *SE* = 0.05, *p* = 0.03), and CAN+ALC-SUD did not differ from either group. For the breathing load, CAN+ALC-SUD showed significantly lower activation than both CTL (*M* = 0.35, *SE* = 0.15, *p* = 0.05) and CAN+ALC-EXP (*M* = 0.40, *SE* = 0.15, *p* = 0.03; see Figure 2).

#### 3.2.2. Follow-Up Correlations

**Figure 3:**
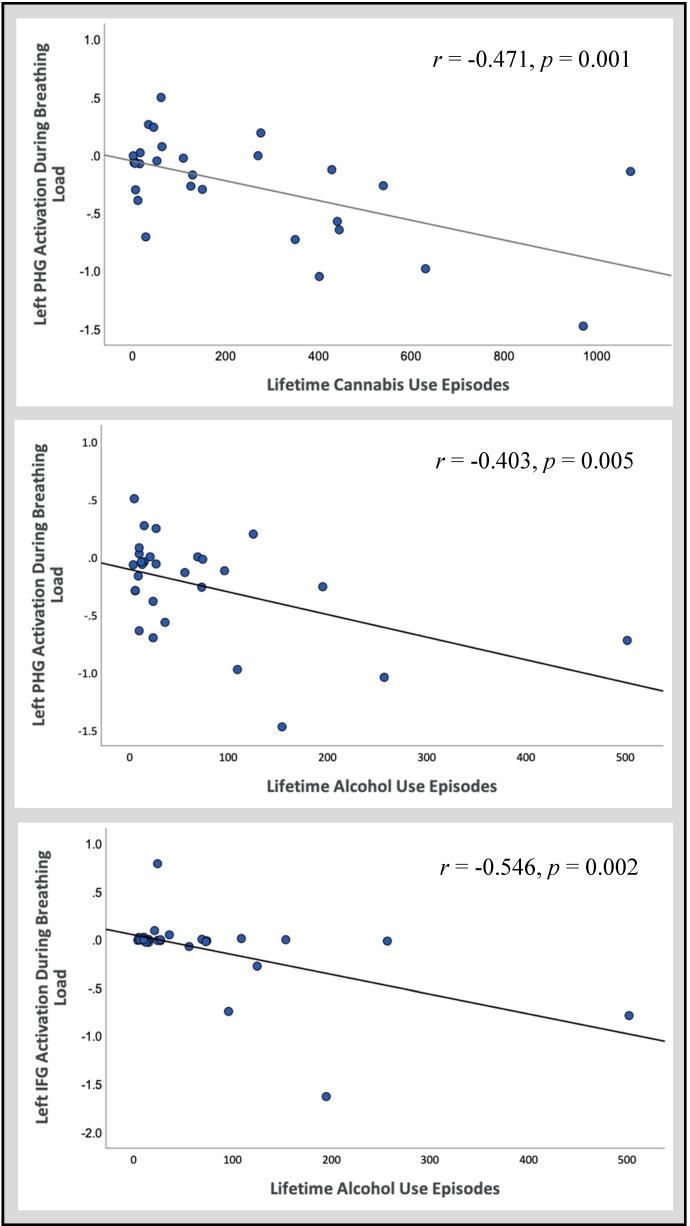
Follow-up correlations between activation in significant regions of interest and reported lifetime episodes of cannabis and alcohol use across all substance users.

Follow-up correlations were conducted within CAN+ALC-SUD and CAN+ALC-EXP between activation in significant regions and lifetime episodes of cannabis and alcohol use. Within the left IFG, activation during the breathing load condition negatively correlated with lifetime episodes of alcohol use (*r* = −0.546, *p* = 0.002, *R*^2^ = 0.298). Within CAN+ALC-SUD and CAN+ALC-EXP, PHG activation during the breathing load condition negatively correlated with lifetime episodes of cannabis (*r* = −0.570, *p* = 0.001, *R*^2^ = 0.325) and alcohol use (*r* = −0.473, *p* = 0.009, *R*^2^ = 0.224; see Figure 3).

## 4. Discussion

The present investigation aimed to examine the role of negative reinforcement in adolescent substance use by pairing a cue reactivity paradigm with an aversive interoceptive probe. It was hypothesized that viewing rewarding substance images would dampen the exaggerated interoceptive response to an aversive probe that has previously been observed in adolescent substance users [27]. Specifically, CAN+ALC-SUD compared to CTL was hypothesized to show: (1) heightened neural activation during the breathing load experience in brain regions involved in interoceptive processing and emotion regulation; (2) heightened neural reward responsivity to substance images; and (3) a decreased interoceptive neural response to the breathing load when paired with substance images. It was also hypothesized that, overall, CAN+ALC-EXP would demonstrate a neural response more similar to CTL than CAN+ALC-SUD.

The hypotheses were partially supported. In relation to hypothesis one, a consistent pattern was observed within the left IFG and the left parahippocampal gyrus, wherein CAN+ALC-SUD exhibited a differential BOLD response to the breathing load compared to CAN+ALC-EXP and CTL (Figure 3; Table 3). Based on previous work demonstrating that adolescent SUD showed an increased response to the breathing load [27], it was hypothesized that CAN+ALC-SUD in the present investigation would also exhibit an increased BOLD response. However, compared to CAN+ALC-EXP and CTL, CAN+ALC-SUD showed greater deactivation during the breathing load. Although this result is inconsistent with previous findings among adolescent substance users [27], it is consistent with previous findings among young adults transitioning from recreational to problematic substance use [39]. A similar pattern was observed in the right amygdala, with CAN+ALC-SUD demonstrating greater deactivation than CAN+ALC-EXP. However, CTL did not differ from either group. Hypothesis two was not supported, as CAN+ALC-SUD did not show a differential reward response to substance images compared to CTL. In line with the lack of an exaggerated reward response to the substance images within CAN+ALC-SUD, viewing these cues did not attenuate the exaggerated interoceptive response exhibited by CAN+ALC-SUD (hypothesis 3). Lastly, it was hypothesized that CAN+ALC-EXP would demonstrate brain responses more similar to CTL than CAN+ALC-SUD; this was partially supported. During the anticipation only condition, CAN+ALC-EXP showed an inconsistent pattern. However, during the breathing load condition, CAN+ALC-EXP did not differ from CTL in the right amygdala, the left IFG, or the left parahippocampal gyrus.

The overall findings suggest that CAN+ALC-SUD experience the aversive breathing load differently than CTL and CAN+ALC-EXP in brain regions implicated in interoception and emotion regulation. However, this observation is in the opposite direction of previous findings. Adolescent SUD has previously shown exaggerated activation rather than deactivation in interoceptive regions when experiencing an aversive breathing load [27]. Additionally, viewing images of alcohol and cannabis did not appear to dampen the blunted interoceptive response seen among CAN+ALC-SUD. This finding would suggest that substance use may not be negatively reinforced by dampening uncomfortable sensations. The pattern of use demonstrated by adolescent substance users (non-treatment-seeking users meeting diagnostic criteria) may not be substantial enough to invoke withdrawal-related symptoms compared to adults who have used heavily for years and/or treatment seekers. Therefore, using in order to relieve uncomfortable sensations may be less common among adolescent users or individuals with less significant use patterns. Future studies should collect subjective ratings of how ‘unpleasant’ and ‘aversive’ participants found the breathing load to be while viewing substance and neutral images separately, as this would provide a clearer understanding of whether or not viewing substance images can contribute to an overall reduction in the aversiveness of the breathing load. Lastly, adolescents with SUD showed amygdala deactivation while experiencing the breathing load but increased activation when anticipating the upcoming load. Previous research has demonstrated that cannabis users exhibit deactivation in the amygdala while viewing emotional images, indicative of altered emotion regulation. This may suggest that the observed group differences in the present study are due to differences in emotion regulation. Although, emotion regulation was not directly assessed in this study, this is a potential avenue for future research.

Interestingly, there were also no significant findings within the insular cortex despite its central role in interoception. This contradicts previous research demonstrating that adolescents meeting criteria for SUD exhibit an increased insular response to the breathing load [27]. It is possible that this lack of insular cortex findings is due to the more stringent thresholding methods employed in the present investigation based on current methodological recommendations for the analysis of fMRI data, as insular activation was present at lower thresholds [51]. Overall, this could suggest that experiencing the breathing load within the context of an experimental manipulation may not be significant enough to elicit a strong insular response among adolescents. Future research should examine whether there is an age-related difference in response to aversive interoceptive perturbations.

The lack of evidence demonstrating any negative reinforcement-related neural response may also be because CAN+ALC-SUD did not find the images rewarding enough, given that an exaggerated reward response was not observed. Altered reward responsivity to substance cues is an established finding among adult substance users [52]. It is possible, given that adolescents with CAN+ALC-SUD typically have significantly less use history than adults with CAN+ALC-SUD, that adolescent reward networks have not yet been altered to show an exaggerated response to substance images. This would suggest that altered reward responsivity is not a predisposition among CAN+ALC-SUD but rather a consequence of use. However, an exaggerated neural response to alcohol and cannabis images in limbic regions has been observed among alcohol-using adolescents and young adults [36,37,53]. A possible reason for our discrepant finding could be differences in characteristics defining each sample. For example, participants in the present study used both alcohol and cannabis. Reward circuitry among alcohol and cannabis users may differ from individuals who only consume alcohol and/or cannabis like those in the previously mentioned investigation [36,37]. Future examination of reward circuitry in single- and multi-substance users with a larger sample could help to elucidate this question.

Clinically, our findings suggest that interventions aiming to improve coping through emotion regulation may not be the most effective for adolescent substance users given the lack of evidence that substance use is driven by negative reinforcement. Alternatively, adolescent substance use may be driven more by positive reinforcement; CAN+ALC-SUD self-reported significantly more motivations for use related to positive, as opposed to negative, reinforcement than CAN+ALC-EXP (Table 1). This aligns with the neurobiological imbalance model, which posits that the development of cognitive control regions is more protracted from childhood to young adulthood, while reward regions follow a curvilinear path of development, with a peak in reward responsivity during adolescence [1,54]. This heightened reward response during adolescence can be seen in reward-processing brain regions (i.e., striatum, insula, anterior cingulate cortex) [55,56,57,58] when anticipating and receiving various types of rewards [59,60]. Behaviorally, this imbalance may contribute to an increase in reward-seeking behaviors, including drug and alcohol experimentation [59] and increased susceptibility to the motivational properties of these substances. This may suggest that interventions aimed at helping adolescents learn alternative ways of experiencing reward may be more effective than those aimed at reducing uncomfortable sensations [61].

Although adolescent substance users report negative reinforcement of substance use, this was not observed using a functional imaging paradigm. As reported above, groups also did not differ in their neural responsivity to the substance images, but this finding may be due to a limitation of study design. The substance images used in the cue reactivity paradigm may not be potent or personally relevant enough to elicit a sufficient neural response to overcome the undesirable impact of the breathing load trials [61,62,63]. In daily life, adolescents may experience uncomfortable interoceptive signals on par with the breathing load experienced within the scanner while the rewarding effects of actual substance use may not be comparable to viewing images. Experimentally administering alcohol and drugs in conjunction with fMRI is an increasingly popular research method that may be more powerful for detecting neural changes related to negative reinforcement [64,65]. Alternatively, creating personalized cue reactivity paradigms using substance-related images from adolescents’ social media accounts may be an alternative method of increasing the valence of the substance cues. Future researchers investigating negative reinforcement principles within adolescent substance users should consider these methods to determine whether a more robust substance cue can elicit neural differences.

An additional limitation of the present study may be the categorization of adolescents based on meeting criteria for CAN+ALC-SUD. The observed correlations between substance use and neural response suggest that future examinations of adolescent substance users may be improved using a dimensional, rather than categorical, approach. Although significant differences in BOLD response to interoceptive stimulation have been observed among adult substance users with and without CAN+ALC-SUD [14,66,67], amount of substance use may be a more differentiating factor than reported CAN+ALC-SUD criteria in young users with comparatively little substance use experience. The small sample size of 13 CAN+ALC-SUD, 16 CAN+ALC-EXP, and 18 CTL also limits the conclusions that can be drawn from the current study and the ability to look at substance-use groups individually (e.g., cannabis vs. alcohol) although comorbid cannabis and alcohol use is common among adolescents [68]. Inclusion of more substance-using adolescents in future studies could help better differentiate between youth who experiment with drugs and alcohol and those who experience more negative consequences related to their use. Lastly, CAN+ALC-SUD and CAN+ALC-EXP significantly differed in the amount of time reported since their last cannabis use. Given that cannabis metabolites can remain in the body for up to three weeks after regular use, it is possible that the differences observed between groups could be due to residual effects in the CAN+ALC-SUD group. Therefore, it is possible that the reported findings are more reflective of the effects of current use and that these differences may resolve with continued abstinence, highlighting another potential avenue for future research.

Despite these limitations, the present study contributes preliminary findings to our overall understanding of substance use in adolescence. The findings further support the hypothesis that interoceptive processing may be altered in substance users. Further, the results suggest that adolescents may not seek substances to reduce negative or uncomfortable sensations, rather use may be driven more by increased sensation-seeking and reward responsivity in adolescence. Examining positive reinforcement in adolescent substance use is an important avenue for future research.

## Figures and Tables

**Table 1:** Characteristics of Substance Use.

**CAN+ALC-** **SUD** **Group Description**	**% Meeting Diagnostic Criteria**		**Diagnostic Criteria Endorsed**				
			***M*** **(*SD*)**	**Min**		**Max**	
THC Use Disorder	92.31		3.42 (1.38)	2		6	
Alcohol Use Disorder	61.54		2.63 (.74)	2		4	
**Substance Use**	**CAN+ALC-SUD** Cannabis/Alcohol Substance Use Disorder	**CAN+ALC-EXP** Cannabis/Alcohol Experimenter	**CTL** Little to No Substance Use	**df**	***t***		***p***
Lifetime Cannabis Use	467.85 (288.05)	39.38 (45.15)	0.17 (0.514)	12.48	−5.31		<0.001
Days Since Last THC Use	18.69 (33.34)	71.69 (82.25)	46.11 (160.19)	20.63	2.35		0.029
Lifetime Alcohol Use	131.92 (131.55)	17.44 (15.87)	0.22 (0.73)	12.28	−3.12		0.009
Days Since Last Alcohol	16.46 (11.67)	45.38 (98.99)	22.22 (66.12)	27	1.04		0.306
Lifetime Alcohol Binge Episode	92.83 (71.90)	7.87 (7.97)	0.11 (0.47)	11.22	−4.07		0.002
Days Since Last Binge	24.70 (24.83)	90.93 (135. 77)	240 (--)	15.38	1.84		0.085
Lifetime Hallucinogen Use	2.69 (3.88)	0.13 (0.50)	--	12.32	−2.37		0.035
Days Since Last Hallucinogen	82.31 (93.58)	9.81 (39.25)	--	15.42	−2.61		0.019
Lifetime Sedative Use	0.77 (1.36)	--	--	12.00	−2.03		0.065
Days Since Last Sedative Use	179.15 (330.97)	--	--	12.00	−1.95		0.075
Lifetime Amphetamine Use	0.31 (1.11)	--	--	12.00	−1.00		0.337
Days Since Last Amphetamine Use	14.46 (52.14)	--	--	12	−1.00		0.337
Lifetime Rx Stimulant Use	1.92 (5.48)	0.06 (0.25)	--	12.04	−1.22		0.245
Days Since Last Rx Stimulant Use	148.23 (297.47)	17.94 (71.75)	--	13.14	−1.54		0.147
Lifetime Cocaine Use	0.92 (1.50)	--	--	12.00	−2.22		0.046
Days Since Last Cocaine Use	55.00 (91.33)	--	--	12.00	−2.17		0.051
Lifetime Ecstasy Use	14.85 (27.65)	--	--	12.00	−1.94		0.077
Days Since Last Ecstasy Use	293.62 (333.72)	--	--	12.00	−3.17		0.008
Lifetime Opiate Use	0.92 (2.75)	1.94 (7.49)	--	27	0.462		0.647
Days Since Last Opiate Use	139.31 (277.92)	26.56 (73.13)	--	13.35	−1.42		0.178
Lifetime Inhalant Use	2.38 (8.30)	--	--	12.00	−1.04		0.321
Days Since Last Inhalant Use	106.00 (259.42)	--	--	12.00	−1.47		0.166
Lifetime Nicotine Use	232.00 (409.19)	4.19 (6.73)	0.56 (2.36)	12.00	−2.01		0.068
Days Since Last Nicotine Use	92.69 (108.66)	130.69 (157.63)	21.94 (93.10)	26.39	0.766		0.451

**Table 2:** Sample Characteristics.

	CAN+ALC-SUD Cannabis/Alcohol Substance Use Disorder	CAN+ALC-EXP Cannabis/Alcohol Experimenter	CTL Little to No Substance Use			
***Demographics***	***M*** **(*SD*)**	***M*** **(*SD*)**	***M*** **(*SD*)**	**df**	***F***	***p***
Age (in years)	16.62 (0.51)	16.69 (0.70)	16.33 (0.77)	2,44	1.27	0.290
Education (in years)	10.46 (0.78)	10.47 (0.83)	10.11 (0.90)	2,43	0.956	0.392
WRAT 4 Verbal IQ	107.31 (14.29)	106.75 (12.37)	112.00 (13.82)	2,44	0.770	0.469
***VAS Ratings***	***M*** **(*SD*)**	***M*** **(*SD*)**	***M*** **(*SD*)**	**df**	***F***	***p***
Unpleasant	5.69 (3.29)	4.63 (2.64)	5.34 (3.49)	2,44	0.432	0.652
Intensity	4.08 (3.47)	2.13 (2.77)	4.41 (2.89)	2,44	2.68	0.08
***Questionnaires***	***M*** **(*SD*)**	***M*** **(*SD*)**	***M*** **(*SD*)**	**df**	***F*** **/*t***	***p***
***MAIA***						
Noticing	2.83 (1.52)	2.75 (1.03)	2.78 (1.19)	2,43	0.016	0.984
Not Distracting	2.14 (0.50)	2.37 (1.12)	2.52 (1.30)	2,43	0.443	0.645
Not Worrying	2.89 (1.43)	2.81 (1.42)	2.70 (1.05)	2,43	0.079	0.925
Attention Regulation	3.17 (.95)	3.45 (0.74)	3.14 (1.15)	2,43	0.494	0.613
Emotional Awareness	3.18 (1.43)	3.04 (1.31)	3.07 (.93)	2,43	0.054	0.948
Self-Regulation	3.10 (1.05)	3.23 (0.90)	3.01 (1.05)	2,43	0.207	0.814
Body Listening	1.36 (1.16)	1.96 (1.44)	1.79 (1.05)	2,43	0.844	0.437
Trusting	3.47 (1.40)	3.73 (1.08)	3.72 (0.92)	2,43	0.231	0.795
***UPPS***						
Lack of Premeditation	2.08 (0.39)	2.18 (0.49)	1.89 (0.42)	2,44	1.92	0.159
Urgency	2.30 (0.66)	2.17 (0.59)	2.06 (0.51)	2,44	0.672	0.516
Sensation Seeking	3.18 (0.29)	3.09 (0.48)	3.03 (0.44)	2,44	0.528	0.594
Lack of Perseverance	2.03 (0.59)	2.13 (0.58)	1.83 (0.34)	2,44	1.53	0.229
***MNRQ***						
Negative Reinforcement	2.85 (2.38)	0.875 (1.63)	--	20.52	2.55	0.019
Positive Reinforcement	11.38 (2.53)	7.25 (3.45)	--	27	3.59	0.001

**Table 3:** fMRI results and between-group comparisons (SUD = CAN+ALC-SUD; EXP = CAN+ALC-EXP).

**GROUP BY INTEROCEPTIVE CONDITION INTERACTION**									
	**R/L**	**Voxels**	**Volume**	**X**	**Y**	**Z**	**BA**	**Anticipation**	**Load**
Amygdala	R	33	2112	28	−9	−30	28	SUD > EXP	EXP > SUD
Inferior Frontal Gyrus	L	28	1792	−13	24	−20	11	--	EXP = CTL > SUD
Posterior Cingulate	R	25	1600	13	−65	16	31	--	EXP = SUD > CTL
Parahippocampal Gyrus	L	21	1344	−24	−7	−19	35	CTL > EXP	CTL = EXP > SUD
**MAIN EFFECT OF INTEROCEPTIVE CONDITION**									
	**R/L**	**Voxels**	**Volume**	**X**	**Y**	**Z**	**BA**	**Condition Effect**	
Cingulate Gyrus	R	3141	201024	8	−6	23	24	Load > Anticipation	
Fusiform Gyrus	R	663	42432	40	−12	−24	20	Anticipation > Load	
Superior Frontal Gyrus	R	334	21376	1	4	57	6	Load > Anticipation	
Cingulate Gyrus	L	131	8384	−2	−25	37	31	Load > Anticipation	
Cuneus	R	112	7168	18	−85	28	18	Load > Anticipation	
Thalamus	R	64	4096	6	−18	4		Load > Anticipation	
Declive	L	61	3904	−15	−63	−20		Load > Anticipation	
Middle Frontal Gyrus	L	48	3072	−36	37	28	9	Anticipation > Load	
Middle Occipital Gyrus	R	43	2752	34	−83	9	19	Load > Anticipation	
Anterior Cingulate	L	39	2496	−6	31	15	24	Load > Anticipation	
Precuneus	R	36	2304	5	−43	43	7	Load > Anticipation	
Precentral Gyrus	R	29	1856	18	−26	64	4	Load > Anticipation	
Precentral Gyrus	L	24	1536	−18	−29	63	4	Anticipation > Load	
**MAIN EFFECT OF CUE STIMULUS TYPE**									
	**R/L**	**Voxels**	**Volume**	**X**	**Y**	**Z**	**BA**	**Stimulus Effect**	
Medial Frontal Gyrus	R	43	2752	1	44	30	9	Substance > Comparison	
Anterior Cingulate	L	23	1472	−1	46	8	32	Substance > Comparison	
